# Intelligent kinematic physics engine construction for hyper-redundant cable-driven flexible manipulator

**DOI:** 10.1371/journal.pone.0353108

**Published:** 2026-07-10

**Authors:** Songtao Wang, Peiang Wang, Xueqian Wang, Sisi Chen, Mingwei Liu

**Affiliations:** 1 Jiangxi Province Key Laboratory of Precision Drive and Equipment, Nanchang Institute of Technology, Nanchang, Jiangxi Province, China; 2 Center for Artificial Intelligence and Robotics, Tsinghua Shenzhen International Graduate School, Tsinghua University, Shenzhen, Guangdong Province, China; University of Lagos Faculty of Engineering, NIGERIA

## Abstract

To solve the problems of difficult kinematics modeling and poor real-time performance for a hyper-redundant cable-driven flexible manipulator, a construction method of an intelligent kinematics physics engine based on Bayesian-optimized Long Short-Term Memory (BO-LSTM) network is proposed in this paper. Firstly, the multilevel kinematics equation of the flexible manipulator is established based on the Denavit-Hartenberg (D-H) parameter method, and an analytical model of the forward solution is established. Secondly, the inverse kinematic model of the Newton iteration method is proposed based on the kinematics equation, and the stability of the numerical solution method is proven. Subsequently, the kinematics database is established based on the Monte Carlo method, the analytical model of forward solution and the high-precision numerical model of inverse solution, and the database is visualized. Finally, an intelligent kinematics physics engine is established based on the kinematics database and the BO-LSTM neural network model. This model combines the global search ability of Bayesian optimization and the advantages of LSTM neural network in processing time series data, which provides a new solution for the kinematic modeling of hyper-redundant cable-driven flexible manipulator. The calculation speed of the forward and inverse BO-LSTM models are 0.018 s and 0.017 s, which are faster than the numerical method. The MSEs of the forward and inverse BO-LSTM models are 0.065 and 0.056 respectively, which are lower than those of the BP, RBF and LSTM neural networks. The SimMechanics module in MATLAB is used to simulate the hyper-redundant cable-driven flexible manipulator. The experimental result shows that the intelligent kinematics physics engine based on the BO-LSTM neural network model has high precision and high efficiency in kinematics solution.

## 1. Introduction

The hyper-redundant cable-driven flexible manipulator is categorized as a type of continuous manipulator. Continuous manipulators have advantages such as slender structures, dexterous movements and high environmental adaptability, and can work in complex spaces and unstructured environments. Continuous manipulators are widely applied in medical surgery [1], inspection and maintenance of nuclear power equipment [2], assembly and monitoring of aircraft parts [3–5], disaster rescue [6], etc. Representative manipulators include the cable-driven bionic elephant trunk manipulator developed by Clemson University in the United States [7], the flexible medical manipulator developed by D. Camarillo [8], and the cable-driven discrete rigid structure snake-like manipulator developed by the Siasun Robot Company, W. Xu [9] and L. Tang [10]. T. Liu [11] proposed a hyper-redundant cable-driven flexible manipulator with segmented structure of cable active drive-passive linkages, which can reduce the number of actuators and improve the stiffness and load capacity of the cable-driven manipulator under the conditions that the working space is constant and the curvature is continuous. With the increasing demand for practical engineering, cable-driven flexible manipulators with hyper-redundant degrees of freedom have received increasing attention, and the real-time problem of inverse kinematics is the key problem to be solved first.

Many scholars have studied the kinematics of continuous manipulators. T. Liu [12] studied the forward and inverse kinematics model of a 6-DOF cable-driven continuous manipulator based on the equal curvature method, and proposed a double iteration method to solve the inverse kinematics model quickly. The solving speed is less than 0.2 s. F. Qi [13] established a kinematics model of a 4-DOF continuous manipulator with elastic intermediate constraints by using the geometric constraint method, and the maximum error is only 3.6%. H. Gu [14] established a kinematic model of a cable-driven snake-like continuous manipulator with 16-DOF based on the D-H parameter method. J. Peng [15] established the pose and velocity kinematics model of the hyper-redundant continuous manipulator based on the geometric method and the D-H parameter method. The average position deviation is 0.521 mm, the average attitude deviation is 0.296°, and the average solution efficiency is 0.193 s. B. He [16] established the kinematics model of 10-DOF soft continuous manipulator based on the Euler-Bernoulli beam theory and analytical method, and the experimental verification was carried out on a prototype with a length of 150 mm. The maximum error is no larger than 5 mm. G. Gao [17] studied the recursive kinematics model of 8-DOF continuous manipulator with axial constraints via a modular method, the manipulator can follow the leader motion, the deviation from the path is less than 9.7% and the tip error is no more than 15.6%. G. Niu [18] established the recursive kinematics model of a multi-degree-of-freedom continuous manipulator based on the equal curvature method. The average deviation is 4.55 mm, and the solution efficiency needs to be further optimized. J. Lu [19] transformed the problem of solving nonlinear inverse kinematics into a problem of solving elliptic equations based on the Kepler elliptic method. The solution efficiency is improved by about 100 times, and the fitting error is reduced by 4 orders of magnitude, but the versatility of the method is poor. Considering the nonlinear factors of kinematics, J. Barrientos [20] established a kinematic model of a 10-DOF continuous manipulator by recursive method in the driving space and configuration space. The model error is 0.7%, and the mean time and the maximum execution time increase linearly. M. Jolaei [21] established a kinematics model of an 8-DOF continuous manipulator based on the fully connected neural network regression method, and analyzed the error and convergence speed. The convergence speed is 0.4 s, and the average error is 0.49 ± 0.32 mm.

In summary, the current kinematics solution method for continuous manipulator can analyze the inverse kinematics solution for continuous manipulators with fewer degrees of freedom. However, an analytical solution for a hyper-redundant cable-driven flexible manipulator with hyper-redundant degrees of freedom is difficult to establish, the current numerical method has difficulty obtaining high accuracy, and the real-time performance of calculations is difficult to ensure. To solve the above problems, this paper proposes an intelligent kinematics physics engine method based on the BO-LSTM model for a hyper-redundant cable-driven flexible manipulator. The intelligent kinematics physics engine is a method to establish the kinematics model of hyper-redundant cable-driven flexible manipulator by using kinematics database and machine learning technology. The forward solution analytical model and inverse solution numerical model based on D-H parameter method and Newton numerical iteration method provide a theoretical basis for the kinematics database. The high-precision kinematics database generated based on the Monte Carlo method provides intuitive and rich samples for the training of machine learning models. The BO-LSTM model learns and grasps the kinematics law of the manipulator based on the kinematics database, thereby achieving intelligent prediction and simulation of the kinematics behavior of the hyper-redundant cable-driven flexible manipulator. The BO-LSTM model combines the global search ability of Bayesian optimization and the time series data processing ability of LSTM neural network, which can effectively deal with the highly nonlinear and strong coupling characteristics of the manipulator. The LSTM neural network controls the long-term preservation and short-term discarding of information by the unique gating mechanism, which effectively solves the problem of gradient disappearance of traditional deep learning methods in processing long sequence data and improves the computational efficiency of the model. The Bayesian optimization achieves efficient search for hyperparameters by constructing a probabilistic model of hyperparameters and updating the model with previously evaluated results, which not only greatly reduces the time cost, but also significantly improves the global calculation accuracy of the model. The intelligent kinematics physics engine significantly improves the accuracy and real-time performance of the kinematics model of the manipulator, and lays a solid foundation for the motion control and optimization of the subsequent complex mechanical system.

## 2. Structure and kinematics of flexible manipulator

### 2.1. Equations overall mechanical structure

**Fig 1:**
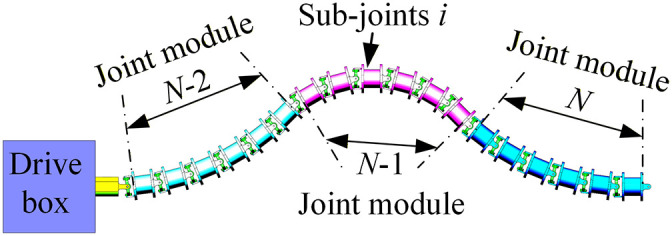
Overall structure of hyper-redundant cable-driven flexible manipulator.

The hyper-redundant cable-driven flexible manipulator has an N-segment joint module with 2N degrees of freedom. The cable tension is controlled by the drive box to drive the joint module movement, as shown in Fig 1. Each joint module contains n sub-joints in series with universal joints, and the adjacent universal joints are constrained by 2 pairs of 8-shaped linkage cables to implement the pitch-yaw angle motion of each sub-joint.

### 2.2. Kinematics modeling of flexible manipulator

**Fig 2:**
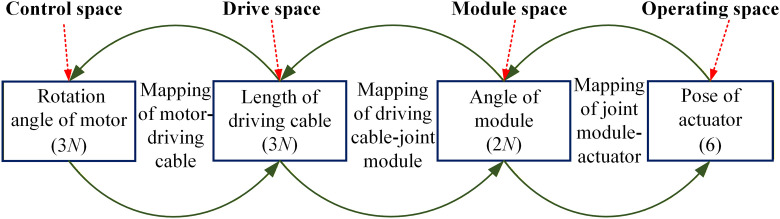
Multilevel kinematic mapping relationship of flexible manipulator.

There are multi-level mapping relationships for the hyper-redundant cable-driven flexible manipulator with N joint modules, including mapping of motor-driving cables, mapping of driving cable-joint modules and mapping of joint module-actuators, as shown in Fig 2. Correspondingly, the kinematics of the flexible manipulator can be gradually decomposed into the following sub-kinematics processes which are the kinematics of the motor-driving cable, the kinematics of the driving cable-joint module and the kinematics of the joint module-actuator.

#### 2.2.1. Kinematics of motor-driving cable.

According to the above analysis, each joint module is driven by three cables, and the stretching motion of each cable is realized by the rotation of the motor. The kinematic model between the *i*th (*i* = 1, 2, 3) motor angle $\theta_{\text{rotor,}n,i}$ of the *n*th (*n* = 1, 2, …, *N*) module and the length of the corresponding driving cable $l_{\text{rotor,}n,i}$ can be obtained by Eq (1).


$$\begin{array}{c} \Delta \theta_{\text{rotor,}n,i}=\frac{2\pi \text{z}}{P}\Delta l_{\text{cable,}n,i} \end{array}$$ (1)

where *P* is the pitch of the ball screw connecting the motor and the driving cable and *Z* is the reduction ratio of the motor speed reducer.

The static model between the motor torque $T_{\text{cable,}n,i}$ and the tension of the driving cable $F_{\text{rotor,}n,i}$ is obtained.


$$\begin{array}{c} F_{\text{rotor,}n,i}=\frac{2\pi \eta \text{z}}{P}T_{\text{cable,}n,i} \end{array}$$ (2)

where η is the pitch of the ball screw.

#### 2.2.2 Kinematics of driving cable-joint module.

**Fig 3:**
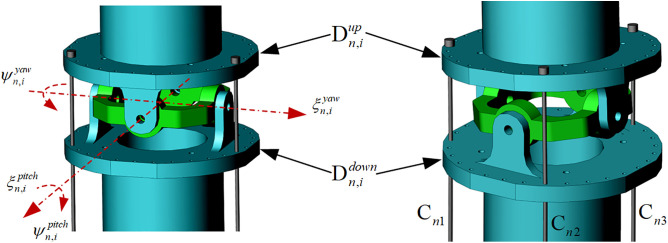
Structure of sub-joint (*n, i*). (a)Zero state. (b)Non-zero state.

The hyper-redundant cable-driven flexible manipulator is composed of N identical linkage joint modules in series, and each joint module has m universal joints of the same size. The sub-joint *i* (*i* = 1, 2, …, *m*) of any joint module n is analyzed, and the structure of sub-joint (*n*, *i*) is shown in Fig 3, and the zero state $\overset{pitch}{\underset{n,i}{\psi }}=\overset{yaw}{\underset{n,i}{\psi }}=0$ and non-zero state $\overset{pitch}{\underset{n,i}{\psi }}\neq 0⋃\overset{yaw}{\underset{n,i}{\psi }}\neq 0$ are shown in Fig 3 (a) and (b), respectively. The sub-joint (*n*, *i*) is a 3-SPS-U parallel mechanism. The upper disk and the lower disk that the three driving cables pass through are respectively represented by $\overset{up}{\underset{n,i}{\text{D}}}$ and $\overset{down}{\underset{n,i}{\text{D}}}$, respectively. The two rotation axes of the universal joint are represented by $\overset{pitch}{\underset{n,i}{\xi }}$ and $\overset{yaw}{\underset{n,i}{\xi }}$, and the corresponding rotation angles are represented by $\overset{pitch}{\underset{n,i}{\psi }}$ and $\overset{yaw}{\underset{n,i}{\psi }}$.

**Fig 4:**
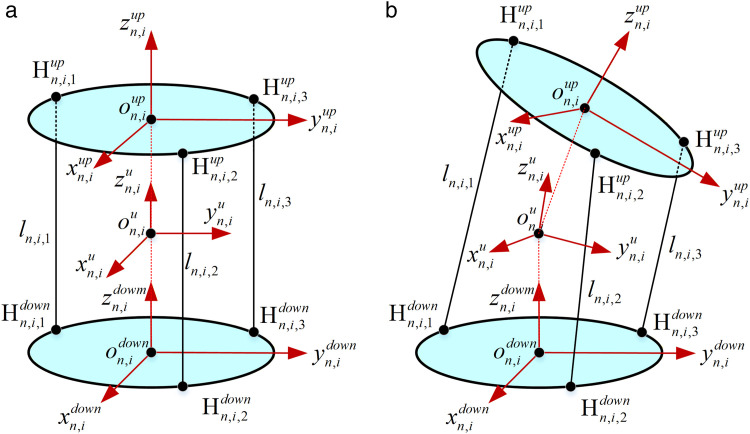
Geometric model of sub-joint (*n, i*). (a) Zero state. (b) Non-zero state.

To establish the kinematic model relating the length of the driving cable and the rotation angle of the sub-joint, the geometric model of sub-joint (n, i) is shown in Fig 4, and the zero state and non-zero state are shown in Fig 4 (a) and (b), respectively. where $\overset{up}{\underset{n,i,k}{\text{H}}}$ and $\overset{down}{\underset{n,i,k}{\text{H}}}$ (k = 1, 2, 3) are the through-hole of the kth driving cable on disks $\overset{up}{\underset{n,i}{\text{D}}}$ and $\overset{down}{\underset{n,i}{\text{D}}}$, respectively. $\overset{up}{\underset{n,i,1}{\text{H}}}\overset{up}{\underset{n,i,2}{\text{H}}}\overset{up}{\underset{n,i,3}{\text{H}}}$ and $\overset{down}{\underset{n,i,1}{\text{H}}}\overset{down}{\underset{n,i,2}{\text{H}}}\overset{down}{\underset{n,i,3}{\text{H}}}$ are the planes formed by the central layers of disks $\overset{up}{\underset{n,i}{\text{D}}}$ and $\overset{down}{\underset{n,i}{\text{D}}}$, respectively. $\overset{up}{\underset{n,i,1}{\text{H}}}\overset{down}{\underset{n,i,1}{\text{H}}}$, $\overset{up}{\underset{n,i,2}{\text{H}}}\overset{down}{\underset{n,i,2}{\text{H}}}$ and $\overset{up}{\underset{n,i,3}{\text{H}}}\overset{down}{\underset{n,i,3}{\text{H}}}$ are three independent driving cables between two disks, with lengths of $l_{n,i,k}$ (k = 1, 2, 3). Two fixed coordinate systems are defined on the two disks. The coordinate system of the upper disk $\overset{up}{\underset{n,i}{\text{D}}}$ is $\overset{up}{\underset{n,i}{o}}-\overset{up}{\underset{n,i}{x}}\overset{up}{\underset{n,i}{y}}\overset{up}{\underset{n,i}{z}}$, and the coordinate system of the lower disk $\overset{up}{\underset{n,i}{\text{D}}}$ is $\overset{down}{\underset{n,i}{o}}-\overset{down}{\underset{n,i}{x}}\overset{down}{\underset{n,i}{y}}\overset{down}{\underset{n,i}{z}}$. The origin of the two coordinate systems is located at the center of the disk, and the z-axis is perpendicular to the surface of the disk and points to the outside. In the zero state, $\overset{up}{\underset{n,i}{x}}$ // $\overset{up}{\underset{n,i}{x}}$ // $\overset{pitch}{\underset{n,i}{\xi }}$, $\overset{up}{\underset{n,i}{y}}$and $\overset{down}{\underset{n,i}{y}}$ are determined by the right-hand rule. In addition, the fixed coordinate systems $\overset{u}{\underset{n,i}{o}}-\overset{u}{\underset{n,i}{x}}\overset{u}{\underset{n,i}{y}}\overset{u}{\underset{n,i}{z}}$, $\overset{u}{\underset{n,i}{x}}$ and $\overset{u}{\underset{n,i}{y}}$ are established at the center of the universal joint, which are parallel to the axes $\overset{pitch}{\underset{n,i}{\xi }}$ and $\overset{yaw}{\underset{n,i}{\xi }}$. The origin is located at the intersection of the axes $\overset{pitch}{\underset{n,i}{\xi }}$ and $\overset{yaw}{\underset{n,i}{\xi }}$, which is the center point of the universal joint, and $\overset{u}{\underset{n,i}{z}}$ is determined by the right-hand rule.

The homogeneous transformation matrix from the coordinate system $\overset{down}{\underset{n,i}{o}}-\overset{down}{\underset{n,i}{x}}\overset{down}{\underset{n,i}{y}}\overset{down}{\underset{n,i}{z}}$ to the coordinate system $\overset{u}{\underset{n,i}{o}}-\overset{u}{\underset{n,i}{x}}\overset{u}{\underset{n,i}{y}}\overset{u}{\underset{n,i}{z}}$ can be obtained Eq (3).


$$\begin{array}{c} \begin{array}{c} \overset{down}{\underset{u}{\;}}A_{n,i}=Trans\left(0,0,d\right)Rot\left(\overset{u}{\underset{n,i}{x}},\overset{pitch}{\underset{n,i}{\psi }}\right)\\ =\left[\begin{array}{cccc} @\;cccc@\;1 & 0 & 0 & 0\\ 0 & c_{\overset{pitch}{\underset{n,i}{\psi }}} & -s_{\overset{pitch}{\underset{n,i}{\psi }}} & 0\\ 0 & s_{\overset{pitch}{\underset{n,i}{\psi }}} & c_{\overset{pitch}{\underset{n,i}{\psi }}} & d\\ 0 & 0 & 0 & 1 \end{array}\right] \end{array} \end{array}$$ (3)

where Trans() is the translation function and Rot() is the rotation function. $c_{\overset{pitch}{\underset{n,i}{\psi }}}=cos\left(\overset{pitch}{\underset{n,i}{\psi }}\right)$, $s_{\overset{pitch}{\underset{n,i}{\psi }}}=sin\left(\overset{pitch}{\underset{n,i}{\psi }}\right)$.

The homogeneous transformation matrix from the coordinate system $\overset{u}{\underset{n,i}{o}}-\overset{u}{\underset{n,i}{x}}\overset{u}{\underset{n,i}{y}}\overset{u}{\underset{n,i}{z}}$ to the coordinate system $\overset{up}{\underset{n,i}{o}}-\overset{up}{\underset{n,i}{x}}\overset{up}{\underset{n,i}{y}}\overset{up}{\underset{n,i}{z}}$ can be obtained Eq (4).


$$\begin{array}{c} \begin{array}{c} \overset{up}{\underset{u}{\;}}A_{n,i}=Trans\left(0,0,d\right)Rot\left(\overset{u}{\underset{n,i}{x}},\overset{yaw}{\underset{n,i}{\psi }}\right)\\ =\left[\begin{array}{cccc} @\;cccc@\;1 & 0 & 0 & 0\\ 0 & c_{\overset{yaw}{\underset{n,i}{\psi }}} & -s_{\overset{yaw}{\underset{n,i}{\psi }}} & 0\\ 0 & s_{\overset{yaw}{\underset{n,i}{\psi }}} & c_{\overset{yaw}{\underset{n,i}{\psi }}} & d\\ 0 & 0 & 0 & 1 \end{array}\right] \end{array} \end{array}$$ (4)

where $c_{\overset{yaw}{\underset{n,i}{\psi }}}=cos\left(\overset{yaw}{\underset{n,i}{\psi }}\right)$, $s_{\overset{yaw}{\underset{n,i}{\psi }}}=sin\left(\overset{yaw}{\underset{n,i}{\psi }}\right)$.

Based on Eq (3) and (4), the homogeneous transformation matrix from coordinate system $\overset{up}{\underset{n,i}{o}}-\overset{up}{\underset{n,i\text{\textasciitilde{}}}{x}}\overset{up}{\underset{n,i}{y}}\overset{up}{\underset{n,i}{\text{\textasciitilde{}}z}}$ to coordinate system $\overset{down}{\underset{n,i}{o}}-\overset{down}{\underset{n,i}{x}}\overset{down}{\underset{n,i}{\text{\textasciitilde{}}y}}\overset{down}{\underset{n,i}{\text{\textasciitilde{}}z}}$ can be obtained Eq (5).


$$\begin{array}{c} \overset{down}{\underset{up}{\;}}A_{n,i}\;\overset{down}{\underset{u}{=\;}}{A_{n,iup}}^{\;\;\;\;\;u}\;\;A_{n,i} \end{array}$$ (5)

According to the physical structure, the through-holes of the driving cables $\overset{up}{\underset{n,i,k}{\text{H}}}$ and $\overset{down}{\underset{n,i,k}{\text{H}}}$ can be obtained Eq (6).


$$\begin{array}{c} \left\{ \begin{array}{c} @\;c@\;\overset{up}{\underset{n,i,k}{h}}\text{=}\left[\begin{array}{cccc} @\;cccc@\;\rho c_{\overset{up}{\underset{n,i,k}{\beta }}} & \rho s_{\overset{up}{\underset{n,i,k}{\beta }}} & 0 & 1 \end{array}\right]\\ \overset{down}{\underset{n,i,k}{h}}\text{=}\left[\begin{array}{cccc} @\;cccc@\;\rho c_{\overset{down}{\underset{n,i,k}{\beta }}} & \rho s_{\overset{down}{\underset{n,i,k}{\beta }}} & 0 & 1 \end{array}\right] \end{array}\right. \end{array}$$ (6)

where ρ is the radius of the circle formed by the uniform distribution of the through-holes on the upper and lower disks. $\overset{up}{\underset{n,i,k}{\beta }}$ is the angle between $\overset{up}{\underset{n,i}{o}}\overset{up}{\underset{n,i,k}{H}}$ and axis $\overset{up}{\underset{n,i}{x}}$. $\overset{down}{\underset{n,i,k}{\beta }}$ is the angle between $\overset{down}{\underset{n,i}{o}}\overset{down}{\underset{n,i,k}{H}}$ and axis $\overset{down}{\underset{n,i}{x}}$. $c_{\overset{up}{\underset{n,i,k}{\beta }}}=cos\left(\overset{up}{\underset{n,i,k}{\beta }}\right)$, $s_{\overset{up}{\underset{n,i,k}{\beta }}}=sin\left(\overset{up}{\underset{n,i,k}{\beta }}\right)$. $c_{\overset{down}{\underset{n,i,k}{\beta }}}=cos\left(\overset{down}{\underset{n,i,k}{\beta }}\right)$, $s_{\overset{down}{\underset{n,i,k}{\beta }}}=sin\left(\overset{down}{\underset{n,i,k}{\beta }}\right)$.

The coordinates of the through-holes $\overset{up}{\underset{n,i,k}{\text{H}}}$ under $\overset{down}{\underset{n,i}{o}}-\overset{down}{\underset{n,i}{x}}\overset{down}{\underset{n,i}{y}}\overset{down}{\underset{n,i}{z}}$ can be obtained Eq (7).


$$\begin{array}{c} \overset{up\to down}{\underset{n,i,k}{h}}\overset{down}{\underset{up}{\;=\;}}A_{n,i}\overset{up}{\underset{n,i,k}{h}} \end{array}$$ (7)

Based on Eq (3) to (7), the length of the three driving cables between the two disks at the sub-joint (*n*, *i*) of the joint *n* can be obtained Eq (8).


$$\begin{array}{c} l_{n,i,k}=\|\overset{down}{\underset{n,i,k}{h}}\overset{up\to down}{\underset{n,i,k}{h}}\| \end{array}$$ (8)

The length of the driving cable between the other two disks of joint *n* can be calculated and the total length of the *k*th cable of the driving cable of the joint module can be obtained Eq (9).


$$\begin{array}{c} f_{n,k}\left(\overset{pitch}{\underset{n}{\theta }},\overset{yall}{\underset{n}{\theta }}\right)=\sum_{i=1}^{\text{4}}l_{n,i,k}\left(\overset{pitch}{\underset{n,i}{\psi }},\overset{yall}{\underset{n,i}{\psi }}\right) \end{array}$$ (9)

where *m* is the number of sub-joints in the joint module.

$\overset{pitch}{\underset{n}{\theta }}$ and $\overset{yall}{\underset{n}{\theta }}$ are the rotation angles of the joint module. The relationship between the rotation angles $\overset{pitch}{\underset{n,i}{\psi }}$ and $\overset{yall}{\underset{n,i}{\psi }}$ of the sub-joints can be obtained Eq (10).


$$\begin{array}{c} \left[\begin{array}{c} @\;c@\;\overset{pitch}{\underset{n}{\theta }}\\ \overset{yall}{\underset{n}{\theta }} \end{array}\right]=\left[\begin{array}{c} @\;c@\;\sum_{i=1}^{m}\overset{pitch}{\underset{n,i}{\psi }}\mathrm{\backslash vspace}1mm\\ \sum_{i=1}^{m}\overset{yall}{\underset{n,i}{\psi }} \end{array}\right] \end{array}$$ (10)

The mapping from the rotation angle of the joint module to the length of the driving cable is established Eq (9) and (10). The inverse kinematics problem involves determining the angle of the joint module based on the length of the driving cable. According to the above analysis, the nth joint module is driven by three driving cables. The following equations can be established.


$$\begin{array}{c} \left\{ \begin{array}{c} @\;c@\;f_{n,1}\left(\overset{pitch}{\underset{n}{\theta }},\overset{yall}{\underset{n}{\theta }}\right)=l_{n,1}\\ f_{n,2}\left(\overset{pitch}{\underset{n}{\theta }},\overset{yall}{\underset{n}{\theta }}\right)=l_{n,2}\\ f_{n,3}\left(\overset{pitch}{\underset{n}{\theta }},\overset{yall}{\underset{n}{\theta }}\right)=l_{n,3} \end{array}\right. \end{array}$$ (11)

where *l*_*n*,*k*_ is the total length of the driving cable *k* at each sub-joint in the joint segment According to the previous mechanical structure design, the length of the cable *l*_*n*,*k*_ in the zero state of the joint segment can be obtained Eq (12).


$$\begin{array}{c} l_{n,k}|{\;}_{\left(0,0\right)}=f_{n,k}\left(0,0\right)=2md \end{array}$$ (12)

There are three known quantities and two unknown quantities in Eq (11), and each equation is a nonlinear equation for unknown quantities. Therefore, it cannot be solved analytically.

The homogeneous equations based on Eq (11) can be obtained Eq (13).


$$\begin{array}{c} F_{n,i}\left(\overset{pitch}{\underset{n}{\theta }},\overset{yall}{\underset{n}{\theta }},l_{n,i}\right)=0\left(i=\;1,2,3\right) \end{array}$$ (13)

The least squares solution of Eq (13) can be obtained via the Newton iteration method. The Newton iteration method is a numerical method to solve the roots or extreme values of nonlinear equations. The core of the Newton iteration method is the Taylor’s formula, which approximates the original function with a polynomial near a certain point. The minimum value of the polynomial is taken as a new iteration point, and this process is repeated until the accuracy requirement is satisfied. The proof that the numerical solution can be obtained by the Newton iteration method is as follows.

It is proven that if the domain of the system $F_{n,i}\left(\overset{pitch}{\underset{n}{\theta }},\overset{yall}{\underset{n}{\theta }},l_{n,i}\right)$ is $D\subset R^{n}$, when $l_{n,i}$ is known, $\overset{pitch}{\underset{n}{\theta }}\in D,\overset{yall}{\underset{n}{\theta }}\in D$ satisfies $F_{n,i}{(}^{*}\overset{pitch}{\underset{n}{\theta }}{,}^{*}\overset{yall}{\underset{n}{\theta }})=0$ in the workspace, ${\dot{F}}_{n,i}{(}^{*}\overset{pitch}{\underset{n}{\theta }}{,}^{*}\overset{yall}{\underset{n}{\theta }})$ exist and is continuous on the open neighborhood $S_{\text{0}}\subset D$ of ${\;}^{*}\overset{pitch}{\underset{n}{\theta }}{,}^{*}\overset{yall}{\underset{n}{\theta }}$, and ${\dot{F}}_{n,i}{(}^{*}\overset{pitch}{\underset{n}{\theta }}{,}^{*}\overset{yall}{\underset{n}{\theta }})$ is nonsingular. Therefore, the iterative sequence generated by the Newton iteration method converges super-linearly to the solution of the equation on the closed interval $S\subset S_{\text{0}}$.

The Jacobian matrix can be obtained by the Newton iteration method and Eq (13).


$$\begin{array}{c} {\dot{F}}_{n}=\left[\begin{array}{cc} @\;cc@\;\frac{\partial F_{n1}}{\partial \overset{pitch}{\underset{n}{\theta }}} & \frac{\partial F_{n1}}{\partial \overset{yall}{\underset{n}{\theta }}}\mathrm{\backslash vspace}1.5mm\\ \frac{\partial F_{n2}}{\partial \overset{pitch}{\underset{n}{\theta }}} & \frac{\partial F_{n2}}{\partial \overset{yall}{\underset{n}{\theta }}}\mathrm{\backslash vspace}1.5mm\\ \frac{\partial F_{n2}}{\partial \overset{pitch}{\underset{n}{\theta }}} & \frac{\partial F_{n2}}{\partial \overset{yall}{\underset{n}{\theta }}} \end{array}\right] \end{array}$$ (14)

**Fig 5:**
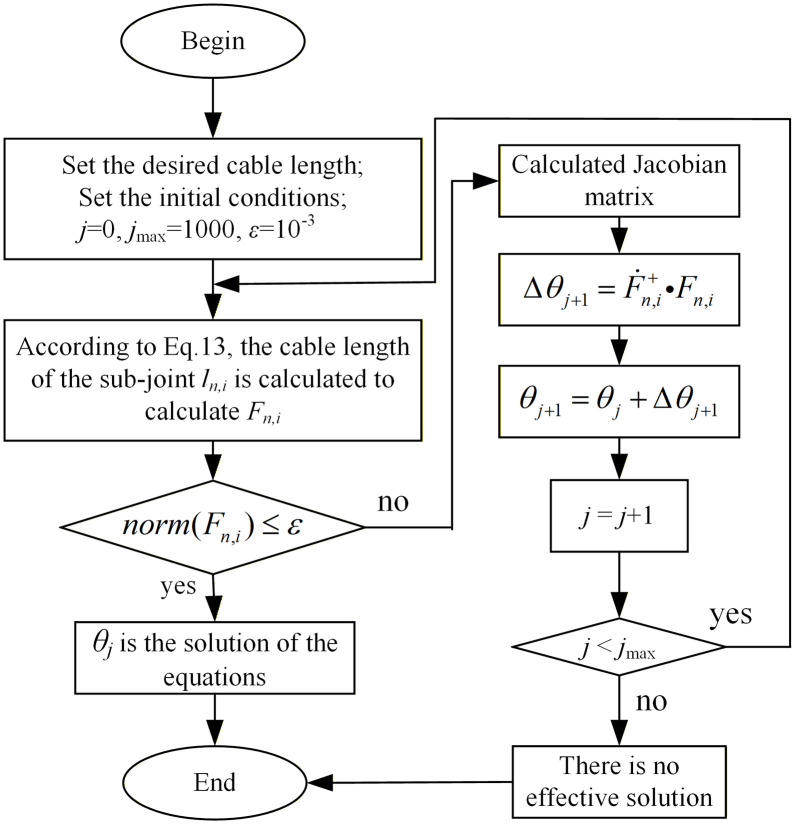
Solution process of sub-joint angle of joint module.

The sub-joint angle of the joint module *n* can be solved by the Newton iteration method, Eq (13) and (14). The solution process is shown in Fig 5, where $\theta =[\overset{pitch}{\underset{n}{\theta }},\overset{yall}{\underset{n}{\theta }}{]}^{T}$, *j* is the number of cycles pointer, and the threshold *ε* is set to 10^−3^.

#### 2.2.3 Kinematics of joint module-actuator.

(1) Forward kinematics

Each joint module of the hyper-redundant cable-driven flexible manipulator is an independent motor unit, and the state of each joint module depends only on its two joint angles. Therefore, the forward kinematics of the joint module-actuator can be expressed as follows.


$$\begin{array}{c} F_{DK}\left(\Theta \right)=\prod_{n=1}^{N}T_{sn} \end{array}$$ (15)

where *T*_*sn*_ is the local transformation matrix of the end of the joint segment *n* relative to its root. Θ is the column vector of the rotation angle $\theta_{\text{1}}\sim \theta_{2\text{N}}$ of all the joint modules of the flexible manipulator. $F_{DK}\left(\Theta \right)$ is the pose matrix of the end of the flexible manipulator.

**Fig 6:**
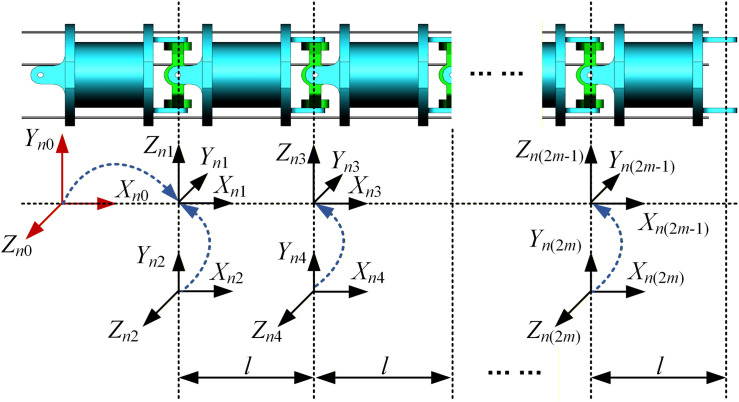
Solution process of sub-joint angle of joint module.

The kinematic analysis of the joint module and the homogeneous transformation matrix *T*_*sn*_ is obtained based on the D-H parameter method. The corresponding D-H coordinate system is established for each joint of any joint module *n*, as shown in Fig 6. The adjacent sub-joints in each joint module have equal rotation angles in parallel coordinates due to the linkage mechanical structure design of the joint module [11]. Therefore, the angles of rotation around *Z*_*n*1_, *Z*_*n*3_,..., *Z*_*n*(2*m*-1)_ are equal in the *n*th joint module, which are expressed as $\overset{yall}{\underset{n}{\theta }}$. The angles of rotation around *Z*_*n*2_, *Z*_*n*4_,..., *Z*_*n*(2*m*)_ are equal, which are expressed as $\overset{pitch}{\underset{n}{\theta }}$.

The forward kinematics equation of the joint module *n* and the homogeneous transformation matrix *T*_*sn*_ of the last sub-joint of the joint module relative to the first sub-joint is as follows.


$$\begin{array}{c} T_{sn}=\overset{\text{1}}{\underset{\text{0}}{T}}\overset{\text{1}}{\underset{\text{2}}{T}}\overset{\text{2}}{\underset{\text{3}}{T}}\overset{\text{3}}{\underset{\text{4}}{T}}\cdots \overset{n(2m-1)}{\underset{n\left(2m\right)}{T}}=f\left(\overset{pitch}{\underset{n}{\theta }},\overset{yall}{\underset{n}{\theta }}\right) \end{array}$$ (16)

where $\overset{i-1}{\underset{i}{T}}$ (i = 1, 2,..., 2m) is the homogeneous transformation matrix between two adjacent coordinate systems of the joint module *n*.

The forward kinematics equation of each joint module can be obtained based on Eq (16), and the homogeneous transformation matrix of the joint module-actuator can be derived.


$$\begin{array}{c} T_{s}=T_{s1}T_{s2}...T_{sn}=\left[\begin{array}{cccc} @\;cccc@\;{\overset{―}{r}}_{11} & {\overset{―}{r}}_{12} & {\overset{―}{r}}_{13} & {\overset{―}{p}}_{x}\\ {\overset{―}{r}}_{21} & {\overset{―}{r}}_{22} & {\overset{―}{r}}_{23} & {\overset{―}{p}}_{y}\\ {\overset{―}{r}}_{31} & {\overset{―}{r}}_{32} & {\overset{―}{r}}_{33} & {\overset{―}{p}}_{z}\\ 0 & 0 & 0 & 1 \end{array}\right] \end{array}$$ (17)

where *T*_*s*_ is a function of variable $\theta_{k}=\left(\overset{pitch}{\underset{\text{1}}{\theta }},\overset{yall}{\underset{\text{1}}{\theta }},\overset{pitch}{\underset{\text{2}}{\theta }},\overset{yall}{\underset{\text{2}}{\theta }},\cdots ,\overset{pitch}{\underset{m}{\theta }},\overset{yall}{\underset{m}{\theta }}\right)$.

According to Eq (17), the position of the end of the manipulator in the *x*-axis, *y*-axis and *z*-axis directions of the absolute coordinate system can be expressed as follows.


$$\begin{array}{c} \overset{―}{p}=\left[\begin{array}{ccc} @\;ccc@\;{\overset{―}{p}}_{x} & {\overset{―}{p}}_{y} & {\overset{―}{p}}_{z} \end{array}\right] \end{array}$$ (18)

The rotation angles *α*, *β* and *γ* of the end of the flexible manipulator around the *x*-axis, *y*-axis and *z*-axis can be expressed as follows.


$$\begin{array}{c} \begin{array}{c} \beta \text{=A }tan2(-{\overset{―}{r}}_{31},\sqrt{\overset{\text{2}}{\underset{11}{\overset{―}{r}}}+\overset{\text{2}}{\underset{21}{\overset{―}{r}}}})\\ \alpha \text{=A }tan2({\overset{―}{r}}_{21}/cos\beta ,{\overset{―}{r}}_{11}/cos\beta )\\ \gamma =\text{A }tan2({\overset{―}{r}}_{32}/cos\beta ,{\overset{―}{r}}_{11}/cos\beta ) \end{array}\} \end{array}$$ (19)

where Atan(*y*, *x*) is the double variable arctangent function. When arctan(*y*/*x*) is calculated, the quadrant of the obtained angle can be determined based on the symbols *x* and *y*.

The forward kinematics of the joint module-actuator can be solved according to Eq (18) and (19), and the end pose of the flexible manipulator can be obtained.

(2) Inverse kinematics

The cable-driven manipulator has multiple inverse kinematics solutions due to its redundant degrees of freedom. The numerical iteration method based on the velocity Jacobian matrix is usually used to solve minimum norm solution. The inverse kinematics equation of the joint module-actuator is constructed based on Eq (17) to (19). When the end pose of rxb is known, the forward kinematics equation is implicitly expressed as follows.


$$\begin{array}{c} \left\{ \begin{array}{c} @\;l@\;f_{\text{1}}=p_{x}\left(\overset{pitch}{\underset{n}{\theta }},\overset{yall}{\underset{n}{\theta }}\right)-p_{x0},f_{\text{2}}=p_{y}\left(\overset{pitch}{\underset{n}{\theta }},\overset{yall}{\underset{n}{\theta }}\right)-p_{y0}\\ f_{\text{3}}=p_{z}\left(\overset{pitch}{\underset{n}{\theta }},\overset{yall}{\underset{n}{\theta }}\right)-p_{z0},f_{\text{4}}=\alpha \left(\overset{pitch}{\underset{n}{\theta }},\overset{yall}{\underset{n}{\theta }}\right)-\alpha_{\text{0}}\\ f_{\text{5}}=\beta \left(\overset{pitch}{\underset{n}{\theta }},\overset{yall}{\underset{n}{\theta }}\right)-\beta_{\text{0}},f_{\text{6}}=\gamma \left(\overset{pitch}{\underset{n}{\theta }},\overset{yall}{\underset{n}{\theta }}\right)-\gamma_{\text{0}} \end{array}\right. \end{array}$$ (20)

The Jacobian matrix is calculated as follows.


$$\begin{array}{c} J_{all}=\left[\begin{array}{ccccc} @\;ccccc@\;\frac{\partial f_{\text{1}}}{\partial \overset{pitch}{\underset{\text{1}}{\theta }}} & \frac{\partial f_{\text{1}}}{\partial \overset{yall}{\underset{\text{1}}{\theta }}} & \cdots & \frac{\partial f_{\text{1}}}{\partial \overset{pitch}{\underset{\text{4}}{\theta }}} & \frac{\partial f_{\text{1}}}{\partial \overset{yall}{\underset{\text{4}}{\theta }}}\mathrm{\backslash vspace}1mm\\ \frac{\partial f_{\text{2}}}{\partial \overset{pitch}{\underset{\text{1}}{\theta }}} & \frac{\partial f_{\text{2}}}{\partial \overset{yall}{\underset{\text{1}}{\theta }}} & \cdots & \frac{\partial f_{\text{2}}}{\partial \overset{pitch}{\underset{\text{4}}{\theta }}} & \frac{\partial f_{\text{2}}}{\partial \overset{yall}{\underset{\text{4}}{\theta }}}\mathrm{\backslash vspace}1mm\\ \vdots & \vdots & \ddots & \vdots & \vdots \\ \frac{\partial f_{\text{5}}}{\partial \overset{pitch}{\underset{\text{1}}{\theta }}} & \frac{\partial f_{\text{5}}}{\partial \overset{yall}{\underset{\text{1}}{\theta }}} & \cdots & \frac{\partial f_{\text{5}}}{\partial \overset{pitch}{\underset{\text{4}}{\theta }}} & \frac{\partial f_{\text{5}}}{\partial \overset{yall}{\underset{\text{4}}{\theta }}}\mathrm{\backslash vspace}1mm\\ \frac{\partial f_{\text{6}}}{\partial \overset{pitch}{\underset{\text{1}}{\theta }}} & \frac{\partial f_{\text{6}}}{\partial \overset{yall}{\underset{\text{1}}{\theta }}} & \cdots & \frac{\partial f_{\text{6}}}{\partial \overset{pitch}{\underset{\text{4}}{\theta }}} & \frac{\partial f_{\text{6}}}{\partial \overset{yall}{\underset{\text{4}}{\theta }}} \end{array}\right] \end{array}$$ (21)

Since the analytical solution of the Jacobian matrix is difficult to calculate, the numerical method is used for implicit solution.


$$\begin{array}{c} \frac{\partial f_{\text{1}}}{\partial \overset{pitch}{\underset{\text{1}}{\theta }}}=\underset{\varepsilon \to 0}{lim}\frac{f_{\text{1}}(\overset{pitch}{\underset{\text{1}}{\theta }}+\varepsilon )-f_{\text{1}}(\overset{pitch}{\underset{\text{1}}{\theta }}-\varepsilon )}{2\varepsilon } \end{array}$$ (22)

**Fig 7:**
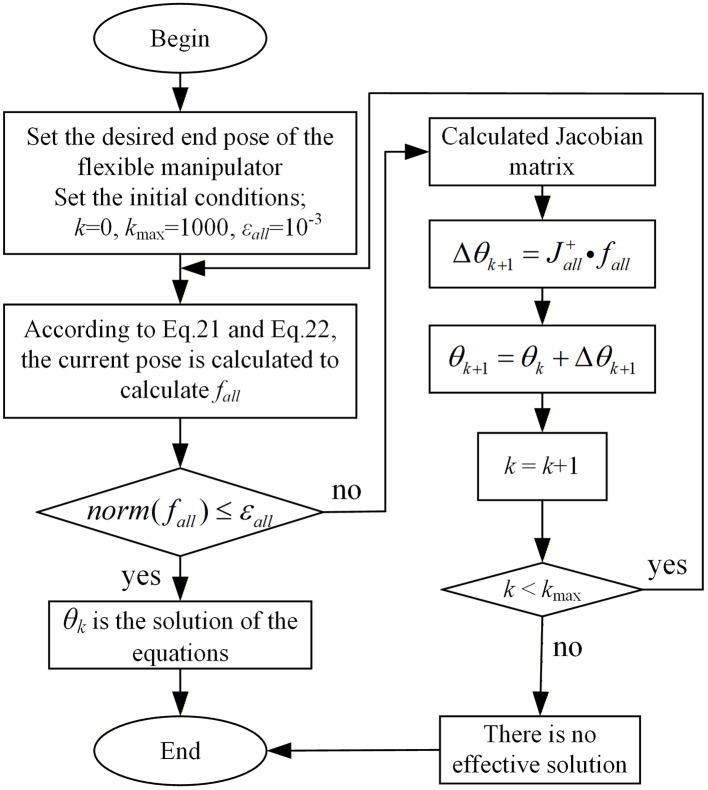
Calculation process of least-energy solution of flexible manipulator.

The whole Jacobian matrix can be numerically solved with high precision and reliability based on Eq (22). The Newton iteration method can be used to complete the solution, and the calculation process is shown in Fig 7.

## 3. Kinematics database based on Monte Carlo method

The dataset is established by a high-precision forward solution, and the artificial intelligence method is used for calculation. It is necessary to construct a high-precision database because only 10% of the randomly generated trajectories are real movement data. There are two methods for establishing the Monte Carlo database, which are the Monte Carlo forward solution database based on the forward kinematics solution method and the Monte Carlo inverse solution database based on the inverse kinematics solution method. Taking the inverse kinematics of the joint module-actuator as an example, the establishment process of establishing two databases is introduced respectively.

### 3.1. Establishment of Monte Carlo forward database

Firstly, in the joint space $\overset{pitch}{\underset{n}{\theta }}$ and $\overset{yall}{\underset{n}{\theta }}$ (*n* = 1, 2, 3, 4), the variable boundary conditions of each joint module are determined based on the physical constraints and task constraints.


$$\begin{array}{c} \left\{ \begin{array}{c} @\;c@\;\overset{pitch}{\underset{n\_min}{\theta }}\leq \overset{pitch}{\underset{n}{\theta }}\leq \overset{pitch}{\underset{n\_max}{\theta }}\mathrm{\backslash vspace}1mm\\ \overset{yall}{\underset{n\_min}{\theta }}\leq \overset{yall}{\underset{n}{\theta }}\leq \overset{yall}{\underset{n\_max}{\theta }} \end{array}\right. \end{array}$$ (23)

where $\overset{pitch}{\underset{n\_min}{\theta }}$ and $\overset{pitch}{\underset{n\_max}{\theta }}$ are the maximum and minimum values of the motion range of the *n*th module pitch degree of freedom, respectively. $\overset{yall}{\underset{n\_min}{\theta }}$ and $\overset{yall}{\underset{n\_max}{\theta }}$ are the maximum and minimum values of the range of motion of the motion range of the *n*th module yall degree of freedom, respectively. Then, the Monte Carlo method is used to generate *K* data points in the motion space of each joint module to form the output data of the Monte Carlo forward solution database. The result of the forward kinematics model is used as the output of the forward solution database.

### 3.2. Establishment of Monte Carlo inverse database

Firstly, the variable boundary conditions of each joint module are determined in the workspace based on the physical and task constraints.


$$\begin{array}{c} \left\{ \begin{array}{c} @\;l@\;p_{x\_min}\leq p_{x}\leq p_{x\_max}\\ p_{y\_min}\leq p_{y}\leq p_{y\_max}\\ p_{z\_min}\leq p_{z}\leq p_{z\_max}\\ \alpha_{min}\leq \alpha \leq \alpha_{max}\\ \beta_{min}\leq \beta \leq \beta_{max}\\ \gamma_{min}\leq \gamma \leq \gamma_{max} \end{array}\right. \end{array}$$ (24)

where *p*_*x*_min_ and *p*_*x*_max_ are the maximum and minimum values of displacement *p*_*x*_ along the *x*-axis respectively. *p*_*y*_min_ and *p*_*y*_max_ are the maximum and minimum values of displacement *p*_*y*_ along the *y*-axis respectively. *p*_*z*_min_ and *p*_*z*_max_ are the maximum and minimum values of displacement *p*_*z*_ along the *z*-axis respectively. *α*_min_ and *α*_max_ are the maximum and minimum values of the rotation angle *α* around the *z*-axis respectively. *β*_min_ and *β*_max_ are the maximum and minimum values of the rotation angle *β* around the *y*-axis respectively. *γ*_min_ and *γ*_max_ are the maximum and minimum values of the rotation angle *γ* around the *x*-axis respectively. Then, the Monte Carlo method is used to generate *Q* data points in the workspace of each joint module to form the input data of the Monte Carlo inverse solution database. The corresponding joint segment angle is calculated based on the inverse solution calculation method to form the output data of the database.

## 4. BO-LSTM neural network mode

The hyper-redundant cable-driven flexible manipulator faces several challenges in kinematics modeling. Firstly, the hyper-redundant cable-driven manipulator has a high degree of freedom, which leads to many parameters to be considered in kinematics model. There is a coupling relationship between the movements of each joint. The movement of one joint may affect the state of other joints, which makes it difficult to establish an accurate kinematic model, and the traditional modeling method is difficult to accurately capture the interaction between joints. Secondly, the Jacobian matrix needs to be calculated when solving the inverse kinematics of the manipulator. For the hyper-redundant manipulator, the dimension of the Jacobian matrix is very high, which leads to a huge amount of calculation when performing inverse kinematics iterative calculations and seriously affects the computational efficiency. Finally, the numerical solution method often faces the contradiction between accuracy and efficiency when the kinematic model of the hyper-redundant cable-driven flexible manipulator is established. On the one hand, in order to obtain high-precision model results, a large number of iterative calculations and fine numerical approximations are required, which leads to a significant reduction in the calculation speed, and it is difficult to meet the application scenarios with high speed requirements such as real-time control. On the other hand, if the numerical solution process is simplified to improve the calculation speed, such as reducing the number of iterations or using approximation algorithms, the accuracy of the model will be sacrificed, and the results cannot accurately reflect the actual motion of the manipulator. To overcome the above difficulties, deep learning provides a new solution. The Long Short-Term Memory (LSTM) neural network is an improved model based on the Recurrent Neural Network (RNN), designed to solve the problems of gradient disappearance and gradient explosion that generally exist in RNNs. LSTM neural network is particularly suitable for establishing real-time data prediction models with large time spans and where data changes are relatively small over time.

There are many hyperparameters in the LSTM neural network model that affect prediction accuracy and regression effect of the model, such as the number of neurons in the LSTM layer, batch size, epoch, and learning rate [22]. These hyperparameters need to be set manually and is difficult to find the optimal solution in a short period of time. To enhance the model’s generalization capability and prediction accuracy, and to determine the most suitable combination of hyperparameters, the Bayesian Optimization (BO) algorithm is introduced into the LSTM neural network. The BO-LSTM model is established to achieve efficient construction of the model and effective improvement of prediction accuracy. The BO algorithm takes the mean square error (MSE) of LSTM model training as the optimization objective, and uses the Gaussian process to construct a surrogate model to approximate the objective function. In each iteration, the BO algorithm intelligently selects the next hyperparameter combination to be evaluated by weighing the exploration and utilization of the acquisition function. As the iteration progresses, the surrogate model and the acquisition function are continuously updated, and the optimal hyperparameter combination that minimizes MSE is gradually approximated. Compared with the traditional method, the BO algorithm only needs to evaluate a small number of hyperparameter combinations in each iteration, which greatly improves the search efficiency and finds the best hyperparameter configuration for the LSTM model, thus significantly reducing the prediction error of the model, improving the prediction accuracy and generalization ability, and making the perform well in time series prediction.

**Fig 8:**
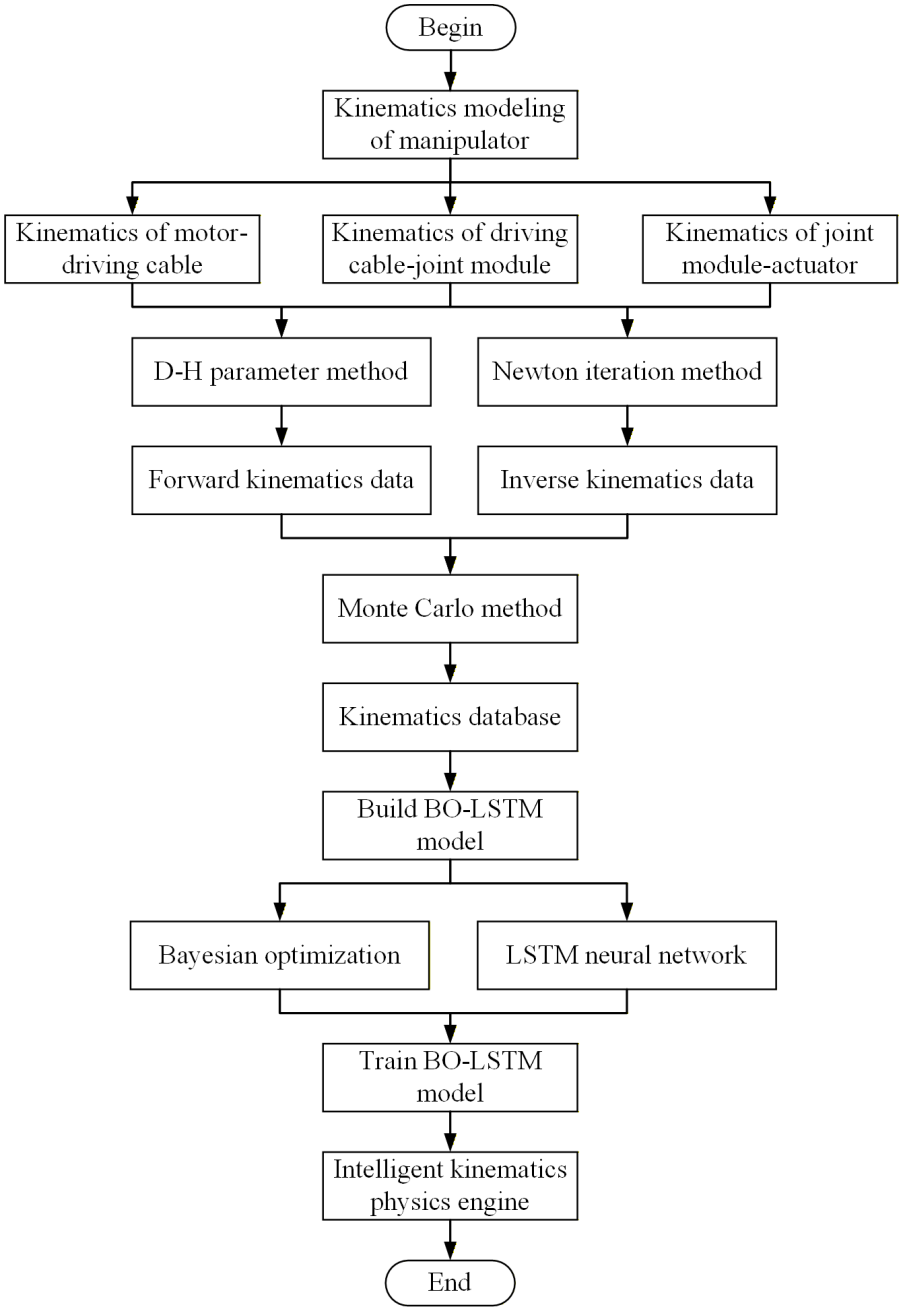
Flowchart of intelligent kinematics physics engine.

At the same time, in order to further improve the computational efficiency of the model and meet the real-time control requirements of the robotic arm, the Early Stopping method is introduced into the LSTM neural network. The training is halted when the performance on the validation set no longer improves, preventing the prediction time of the model from becoming excessively long [23]. To clearly demonstrate the construction process of the intelligent kinematics physics engine for the kinematics modeling of the hyper-redundant cable-driven flexible manipulator based on the BO-LSTM model, the flowchart is shown in Fig 8.

### 4.1. LSTM neural network

**Fig 9:**
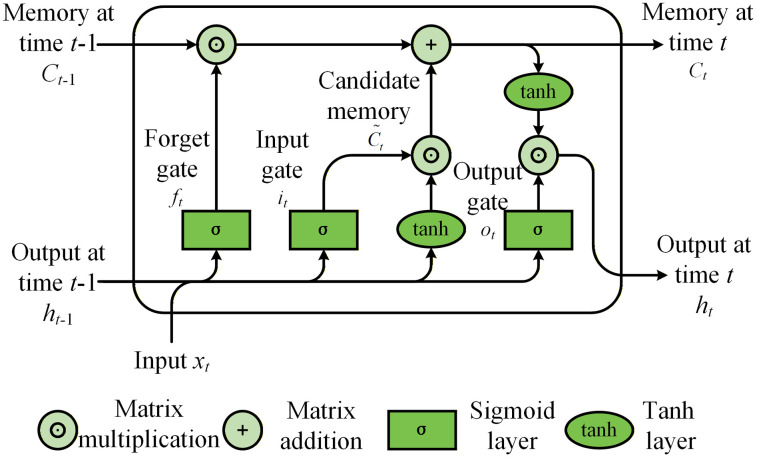
Initial unit structure of LSTM neural network.

The LSTM neural network is a variant of the CNN and is composed of a series of neurons. The LSTM neural network can remember long-term information and avoid the problems of exploding or vanishing [24]. The long-term memory of the LSTM neural network depends on its unit structure, as shown in Fig 9. The unit structure of the LSTM neural network is a gate unit, and the predicted value of the current moment is calculated by controlling the state information of the forget gate, the input gate and the output gate.

where *x*_*t*_ is the input vector of the hidden layer at time *t*. *h*_*t*-1_ is the output vector of the hidden layer at time *t*-1. *C*_*t*-1_ is the state of the hidden layer cells at time *t*-1. it is the control vector of the input gate at time *t*. ${\tilde{C}}_{\text{t}}$ is the candidate vector at time *t*. *o*_*t*_ is the control vector of the output gate at time *t*.

The forward calculation process is as follows. In the first step, how much information is forgotten from the previous unit is determined by the Sigmoid layer of the forget gate. The *f*_*t*_ is a number between 0 and 1 obtained by the Sigmoid function. The number 1 means that the information is completely retained, and the number 0 means that the information is completely discarded. The calculation formula for ft is as follows.


$$\begin{array}{c} f_{t}=\sigma (W_{fh}h_{t-1}+W_{fx}x_{t}+b_{f}) \end{array}$$ (25)

In the second step, whether the input value at the current time is saved to the unit state is determined by the Sigmoid layer of the input gate. The index *i*_*t*_, the candidate vector of the current state ${\tilde{C}}_{\text{t}}$, and calculating the status information of the current state *C*_*t*_ are calculated as follows.


$$\begin{array}{c} i_{t}=\sigma (W_{ih}h_{t-1}+W_{ix}x_{t}+b_{i}) \end{array}$$ (26)

$$\begin{array}{c} {\tilde{C}}_{\text{t}}=tanh(W_{Ch}h_{t-1}+W_{Cx}x_{t}+b_{C}) \end{array}$$ (27)

$$\begin{array}{c} C_{t}=f_{t}C_{t-1}+i_{t}{\tilde{C}}_{\text{t}} \end{array}$$ (28)

In the last step, the unit state to be output is determined by the Sigmoid layer of the output gate. Then, we can calculate *o*_*t*_. The unit state is normalized and then multiplied by the output gate result, and the output value of the current moment is finally obtained.


$$\begin{array}{c} o_{t}=\sigma (W_{oh}h_{t-1}+W_{ox}x_{t}+b_{o}) \end{array}$$ (29)

$$\begin{array}{c} h_{t}=o_{t}tanh\left(C_{t}\right) \end{array}$$ (30)

The predicted value *h*_*t*_ at time *t* can be obtained Eq (30).

### 4.2. BO algorithm

BO algorithm is a global optimization method for finding the extremum of an unknown objective function f(x), which includes a probabilistic surrogate model and Bayesian theorem [25]. The probabilistic surrogate model and the acquisition function are the core of the BO algorithm. The probabilistic surrogate model is used to simulate the distribution of the objective function, and the acquisition function estimates the optimal value based on the posterior distribution. BO algorithm replaces the function f(x) based on probabilistic surrogate model during the initial stages of optimization. The most commonly model is the Gaussian Processes function, which is widely applied in non-linear data fitting, regression, and clustering problems. The Gaussian Processes function is calculated as follows [26].


$$\begin{array}{c} f\left(x\right)\sim \;GP\left(\mu \left(x\right),k\left(x,x^{\prime }\right)\right) \end{array}$$ (31)

$$\begin{array}{c} \mu_{x}=E\left(f\left(x\right)\right) \end{array}$$ (32)

$$\begin{array}{c} k\left(x,x^{\prime }\right)=E\left[\left(f\left(x\right)-\mu \left(x\right)\right)\left(f\left(x^{\prime }\right)-\mu \left(x^{\prime }\right)\right)\right] \end{array}$$ (33)

where μ(x) is the mathematical expectation of f(x). $k\left(x,x^{\prime }\right)$ is the covariance function of *x*. $\mu_{x}$ is the mean function.

The core idea of the Bayesian theorem is to utilize the prior probability distribution of the probabilistic surrogate model, along with the known set of observations obtained through random sampling, to calculate the posterior probability distribution. Based on the posterior distribution, a more reasonable sampling point is selected for the next iteration. The extremum point can be obtained by approximated the modified probabilistic surrogate model to f(x). The Bayesian theorem function is calculated as follows [27].


$$\begin{array}{c} p\left(f|D\right)=\frac{p\left(D|f\right)p\left(f\right)}{p\left(D\right)} \end{array}$$ (34)

Where *f* is an unknown objective function. $D=\left\{\left(x_{\text{1}},y_{\text{1}}\right),\left(x_{\text{2}},y_{\text{2}}\right),\left(x_{\text{3}},y_{\text{3}}),...,(x_{t},y_{t}\right)\right\}$ is the known set of observations, $x_{t}$ is the decision variable, $y_{t}=f\left(x_{t}\right)+\varepsilon_{t}$ is the observation, $\varepsilon_{t}$ is the error of observation. p(f|D) is the posterior probability distribution of f. p(D|f) is the likelihood distribution of *y*. p(f) is the prior probability distribution of *f*. p(D) is the marginal likelihood distribution of *f*.

### 4.3. Design of BO-LSTM neural network model

The BO algorithm is embedded into the LSTM neural network model, and the minimum value of the root mean square error (RMSE) is used as the objective optimization model to solve the optimal hyperparameter combination of the LSTM neural network model, and the hyperparameter combination is output to the prediction model for model training. The BO algorithm can efficiently complete the optimal selection of hyperparameters within the set range of hyperparameters compared with the traditional method of manually selecting hyperparameters in LSTM neural network models, which can not only reduce the time cost, but also improve the robustness and accuracy of the model. In this paper, four hyperparameters need to be optimized in the neural network model, which are the number of neurons in the LSTM layer, the batch size, the epoch, and the learning rate. The interval setting of the BO-LSTM hyperparameters is shown in Table 1.

#### 4.3.1. Design of input layer and output layer.

In the neural network model, the number of neurons in the input layer and the output layer is closely related to the feature dimension of the problem, which ensures that the neural network can accurately capture and process the key information related to the motion of the manipulator, so as to realize the effective prediction and control of the motion state of the manipulator. The number of neurons in the input layer is generally equal to the number of dimensions of the training samples, and the number of neurons in the output layer is equal to the number of the spatial dimensions of the output results [28].

The task of the forward intelligent physical engine is to predict the position and attitude of the end of the manipulator based on the rotation angle of the manipulator joint. The manipulator is composed of 4 joint modules, and each joint module has 2 rotation angles, so there are 8 rotation angles to describe the joint state of the manipulator. The 8 rotation angle parameters constitute the feature dimension of the input data, so the number of neurons in the input layer is 8. Each neuron corresponds to a rotation angle parameter, so that the joint angle information is completely input into the neural network, which provides the basis for subsequent calculation and prediction. The number of neurons in the output layer is 6, which corresponds to the position and attitude of the end of the manipulator, including 3 position coordinates (x, y, z) and 3 attitude angles (Roll, Pitch and Yaw). These 6 parameters can fully describe the position and direction of the end of the manipulator in three-dimensional space. The forward intelligent physics engine can achieve an effective mapping from the joint angle to the end position based on the above input and output settings.

The task of the inverse intelligent physics engine is to reverse the rotation angle of each joint module according to the expected position and attitude of the end of the manipulator, so the number of neurons in the input and output layers is opposite to that of the forward intelligent physics engine. In the inverse intelligent physics engine, the number of neurons in the input layer is 6, which corresponds to the position and attitude of the end of the flexible manipulator, and the number of neurons in the output layer is 8, which corresponds to 8 rotation angles of 4 joint modules of the flexible manipulator.

#### 4.3.2. Design of hidden layer.

The hidden layer of the BO-LSTM neural network includes the LSTM layer and the fully connected layer. The role of the LSTM layer is to process the continuous distance information sequence, and the role of the fully connected layer is to merge the output of each LSTM unit [29]. To obtain favorable prediction performance, the hidden layer of the BO-LSTM neural network model is determined to be 4 layers, of which the first three layers are the LSTM layers and the fourth layer is the fully connected layer.

The number of neurons in the LSTM layer is determined by experience, which can be obtained Eq (35).


$$\begin{array}{c} l=\sqrt{n+m}+a \end{array}$$ (35)

where *l* is the number of neurons in the LSTM layer, *n* is the number of neurons in the input layer, *m* is the number of neurons in the output layer, and *a* is a constant between 1 and 10. The range of neurons in the LSTM layer can be determined to be 5–14 by (35). The total number of neurons in the fully connected layer is determined by the number of outputs.

#### 4.3.3. Activation function and training function.

In deep learning, common activation functions include the Sigmoid function, Tanh function and Relu function. The Sigmoid function and Tanh function are chosen as activation functions based on the special unit structure of BO-LSTM neural network. The Sigmoid function can constrain the amplitude of the data and improve the accuracy of the calculation. The derivative of the Tanh function ranges from 0 to 1, which alleviates the problem of gradient disappearance.

The training functions include the traingdm function, traingdx function and trainlm function. In this paper, the trainlm function is chosen as the training function of LSTM neural network based on the convergence rate and prediction accuracy.

**Table 1:** Interval setting of BO-LSTM hyperparameters.

Hyperparameters	Interval
LSTM layer neurons	[5 14]
Batch size	[200, 800]
Epoch	[200, 300]
Learning rate	[10 3 10 1]

#### 4.3.4. Determination of batch size, epoch and learning rate.

The sample is divided into multiple batches before training, which can improve the memory utilization. The range of batch size of the BO-LSTM neural network model is 200–800 based on the computation space. In the training process, the error value decreases slightly after 200 epochs, and reaches the lowest value after 500 epochs. Therefore, the range of epochs is set to 200–500 in this paper. Whether the neural network can obtain the global optimum is determined by the learning rate [30]. The range of the initial learning rate is set to 10^−3^ to 10^−1^ based on repeated experiments, the convergence of the training process and the training time.

### 4.4. Operation of BO-LSTM neural network

#### 4.4.1. Data treatment.

**Fig 10:**
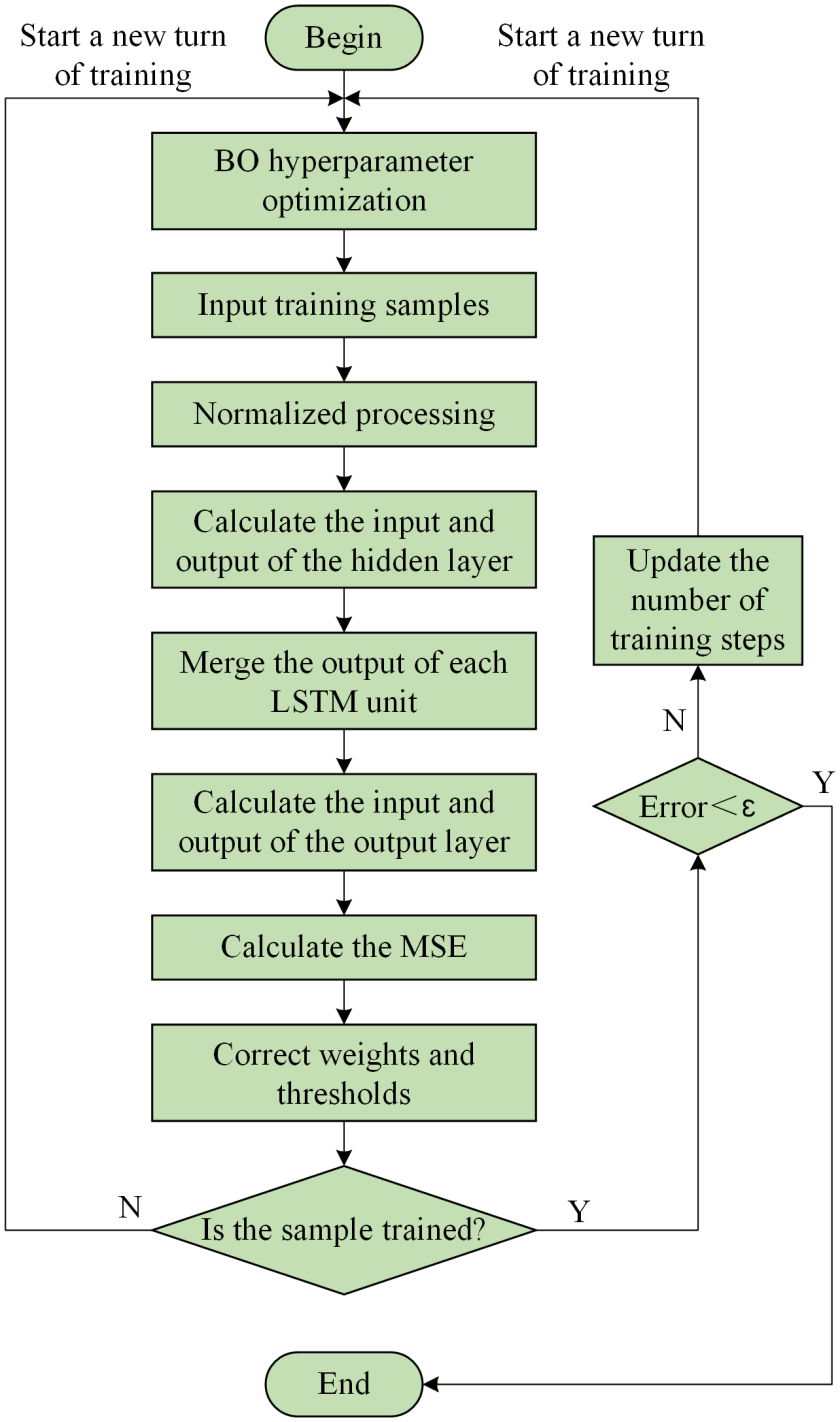
Algorithm process of BO-LSTM model.

The forward kinematics model is used to randomly generate 2000 sets of data. To better evaluate the performance and generalization ability of the model, we divide the dataset into training set, validation set and test set in the proportion of 80%, 15% and 5% respectively. The training set is used to fully train the model, and the validation set is used to adjust the hyperparameters of the model during the training process to prevent overfitting. The test set is used to evaluate the predictive performance of the model. This division method can ensure that the model is fully trained and verified at different stages, thereby improving the reliability and accuracy of the model in practical applications. The algorithm flow of the BO-LSTM neural network is shown in Fig 10.

The dataset is normalized before training [31]. The min-max method is a commonly used normalization method.


$$\begin{array}{c} \overset{―}{x}=\frac{x-x_{min}}{x_{max}-x_{min}} \end{array}$$ (36)

where *x*_max_ and *x*_min_ are the maximum and minimum values in the dataset, respectively.

#### 4.4.2. Error metrics.

To evaluate the effectiveness of the prediction model accurately, the mean square error (MSE), mean relative error (MRE), mean absolute error (MAE) and coefficient of determination (R^2^) are used to evaluate the prediction effect of the model [32]. The calculation equations of the error metrics are as follows.


$$\begin{array}{c} M\text{S}E=\frac{\text{1}}{n_{all}}\sum_{i=1}^{n_{all}}(q_{i}-g(x_{i}){)}^{\text{2}} \end{array}$$ (37)

$$\begin{array}{c} M\text{R}E=\frac{\text{1}}{n_{all}}\sum_{i=1}^{n_{all}}\left|\frac{q_{i}-g\left(x_{i}\right)}{q_{i}}\right| \end{array}$$ (38)

$$\begin{array}{c} M\text{A}E=\frac{\text{1}}{n_{all}}\sum_{i=1}^{n_{all}}\left|q_{i}-g\left(x_{i}\right)\right| \end{array}$$ (39)

$$\begin{array}{c} R^{\text{2}}=1-\frac{\sum_{i=1}^{n_{all}}(q_{i}-g(x_{i}){)}^{\text{2}}}{\sum_{i=1}^{n_{all}}(q_{i}-\overset{―}{q}{)}^{\text{2}}} \end{array}$$ (40)

where *g*(*x*_*i*_) is the predicted joint angle. *q*_*i*_ is the actual joint angle. $\overset{―}{q}$ is the average value of the actual joint angle. *n*_all_ is the number of samples.

In order to more fully explain the credibility and accuracy level of the BO-LSTM model, the confidence interval estimation of the prediction results of the joint angle of the manipulator is carried out. The narrow confidence interval indicates that the model has less uncertainty in the prediction results, the predicted values are more concentrated, and the prediction accuracy of the model is higher. This shows that the model can better capture the rules and trends in the data, and is more sensitive and accurate to the changes in the input data. Assuming that the prediction results of BP, RBF, LSTM and BO-LSTM models for the joint angle of the manipulator obey the normal distribution *X* ~ *N* (*μ*, *σ*^2^), and the credibility is 95%, the confidence interval solution formula of the mean value of the prediction results is as follows.


$$\begin{array}{c} CI=\overset{―}{g}\left(x_{i}\right)\pm Z\times \frac{s}{\sqrt{n_{all}}} \end{array}$$ (41)

Where $\overset{―}{g}\left(x_{i}\right)$ is the average of the predicted results. *s* is the sample standard deviation of the prediction results. *Z* is the critical value of the standard normal distribution. For the 95% confidence level, *Z* = 1.96.

## 5. Simulation verification

### 5.1. Construction of kinematics database

#### 5.1.1. Simulation of Monte Carlo forward database.

**Fig 11:**
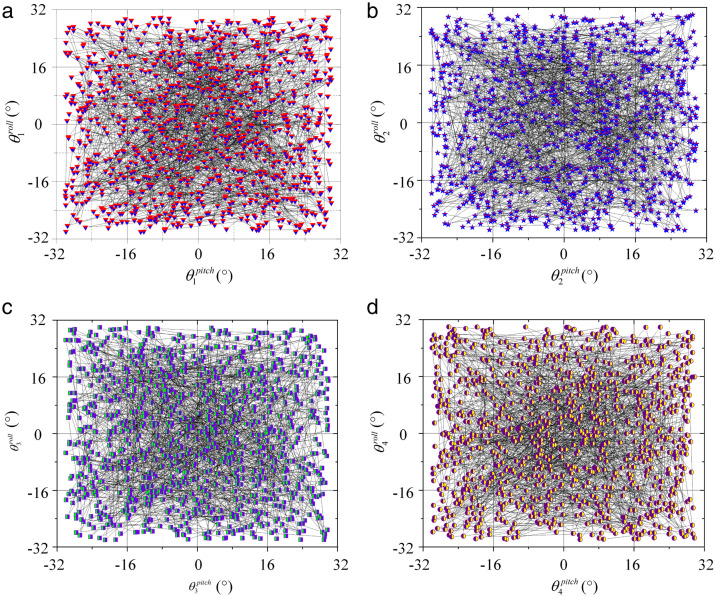
Database visualization of joint module. (a) Database of $\overset{pitch}{\underset{1}{\theta }}-\overset{roll}{\underset{1}{\theta }}$. (b) Database of $\overset{pitch}{\underset{2}{\theta }}-\overset{roll}{\underset{2}{\theta }}$. (c) Database of $\overset{pitch}{\underset{3}{\theta }}-\overset{roll}{\underset{3}{\theta }}$. (d) Database of $\overset{pitch}{\underset{4}{\theta }}-\overset{roll}{\underset{4}{\theta }}$.

**Fig 12:**
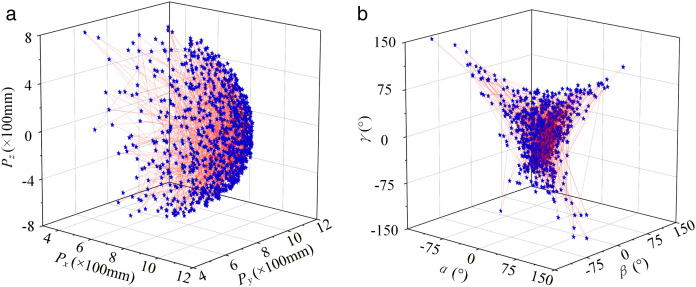
Database visualization of end pose. (a) Position database. (b) Attitude database.

The Monte Carlo method approximates the problem by a large number of random sample points. The larger the number of sample points, the higher the stability and accuracy of the results, but the computational cost will increase accordingly. Through experience and experiments, we found that when *K* = 103, the consumption of computing resources can be reduced on the basis of ensuring the stability of model training and the accuracy of solution. The structural parameters of the continuous manipulator are *n* = 4, *K* = 103, $\overset{pitch}{\underset{n\_min}{\theta }}$ = −30°, $\overset{pitch}{\underset{n\_max}{\theta }}$ = 30°, $\overset{yall}{\underset{n\_min}{\theta }}$ = 30°, and $\overset{yall}{\underset{n\_max}{\theta }}$ = 30°. The pitch and roll angle databases of the four joints of the manipulator are shown in Fig 11, and the corresponding position and attitude databases of the end of the manipulator are shown in Fig 12. The experimental hardware used is an 11th Gen Intell i7-11800H CPU with 32GB of RAM. The growing time of the database is 1.5 s.

According to the results of the database visualization of the joint module, the above results reflect the random uniform distribution characteristics of the data generated by the Monte Carlo method. According to the results of the database visualization of end poses, the database based on the Monte Carlo method has the advantages of having a relatively concentrated data distribution, all the data being valid, having no data beyond the boundary, and having no correlation between the data. Therefore, the database of end poses has the characteristics of random assortment.

#### 5.1.2. Simulation of Monte Carlo inverse database.

**Fig 13:**
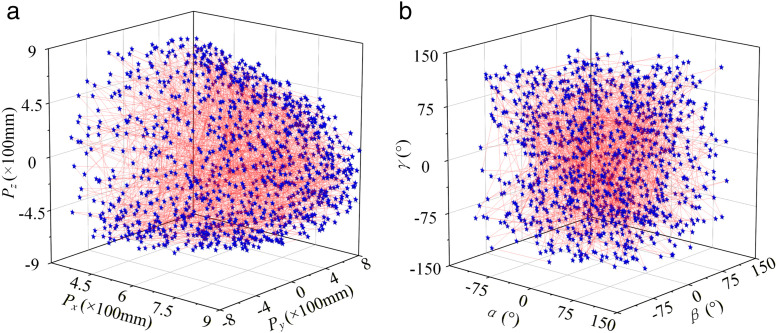
Database visualization of end pose. (a) Position database. (b) Attitude database.

**Fig 14:**
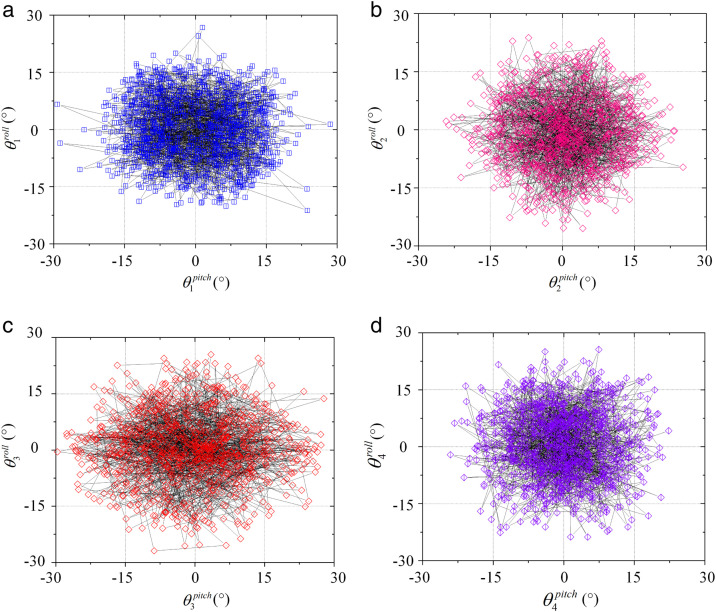
Database visualization of joint module. (a) Database of $\overset{pitch}{\underset{1}{\theta }}-\overset{roll}{\underset{1}{\theta }}$. (b) Database of $\overset{pitch}{\underset{2}{\theta }}-\overset{roll}{\underset{2}{\theta }}$. (c) Database of $\overset{pitch}{\underset{3}{\theta }}-\overset{roll}{\underset{3}{\theta }}$. (d) Database of $\overset{pitch}{\underset{4}{\theta }}-\overset{roll}{\underset{4}{\theta }}$.

The structural parameters of the flexible manipulator are *n* = 4, *Q* = 10^3^, $\overset{pitch}{\underset{n\_min}{\theta }}$ = −30°, $\overset{pitch}{\underset{n\_max}{\theta }}$ = 30°, $\overset{yall}{\underset{n\_min}{\theta }}$ = 30°, and $\overset{yall}{\underset{n\_max}{\theta }}$ = 30°. The value of *Q* is based on experience and experiments. The initial conditions for constructing the inverse solution are as follows. *p*_*x*_min_ = −800 mm, *p*_*x*_max_ = 800 mm, *p*_*y*_min_ = −800 mm, *p*_*y*_max_ = 800 mm, *p*_*z*_min_ = − 800 mm, *p*_*z*_max_ = 800 mm, *α*_min_ = −150 mm, *α*_max_ = 150 mm, *β*_min_ = −150 mm, *β*_max_ = 150 mm, *γ*_min_ = −150 mm, *γ*_max_ = 150 mm. The position and attitude databases of the end of the manipulator are shown in Fig 13, and the corresponding pitch and roll angle databases of the four joints of the manipulator are shown in Fig 14. The experimental hardware used is an 11th Gen Intell i7-11800H CPU with 32GB of RAM. The generation time of the inverse database is 898.66 s.

The database of the joint module established by the Monte Carlo method has the advantages of uniform data distribution, all the data are valid and the adjacent data are discontinuous. The above results reflect the random uniform distribution characteristics of the data generated by the Monte Carlo method. The inverse database shown in Fig 14 shows that the phenomenon of data concentration in the four groups of databases, and the data lose their Monte Carlo characteristics after the inverse solution. The main reason is the super-strong nonlinear coupling characteristics of the continuous manipulator, which make the end pose data of the composite Monte Carlo characteristics become non-uniform.

The simulation of Monte Carlo forward database and inverse database shows that the forward database is reachable workspace, and the inverse database is the minimum energy database and sensitivity space. The results show that the data in the forward database and in the inverse database calculated by the continuous manipulator will have concentrated distortion after the super-strong nonlinear geometric influence of the continuous manipulator.

### 5.2. Kinematic intelligent engine construction based on BO-LSTM

#### 5.2.1 Error analysis of intelligent physics engine.

**Fig 15:**
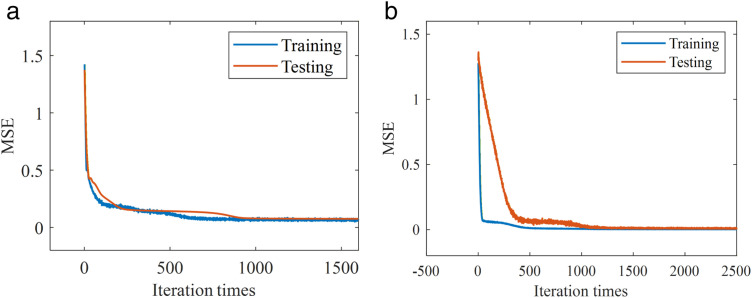
Error loss in training and testing processes. (a) Forward error loss. (b) Inverse error loss.

The training parameters of the forward intelligent physics engine can be set as follows based on the hyperparameter optimization of BO algorithm. The batch size is 400, the number of neurons in the LSTM layer is 6, the learning rate is 0.01, and the number of epochs is 400. The training parameters of the inverse intelligent physics engine can be configured based on the inverse solution database. The batch size is 320, the number of neurons in the LSTM layer is 12, the learning rate is 0.001, and the number of epochs is 500. The errors in the training and testing processes are shown in Fig 15.

**Fig 16:**
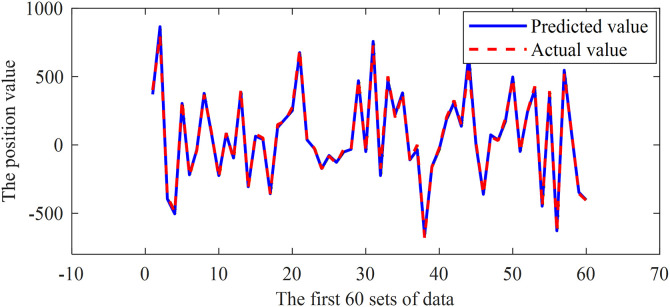
Prediction effect of forward intelligent physics engine.

**Fig 17:**
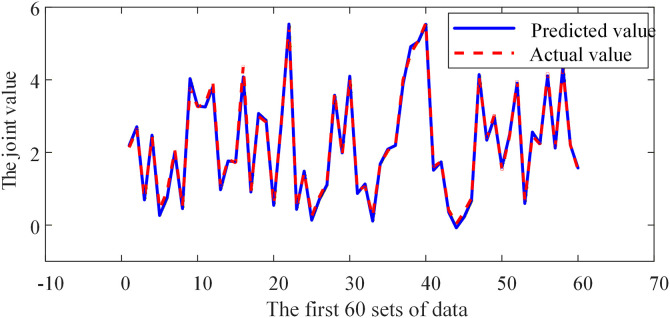
Prediction effect of inverse intelligent physics engine.

After the training of the BO-LSTM neural network model is completed, the testing set is used to test the accuracy of the model. Taking the first position of the end of the flexible manipulator as an example, the prediction effect of the forward intelligent physics engine is shown in Fig 16, and the prediction effect of the inverse intelligent physics engine is shown in Fig 17. The solid line is the predicted value and the dashed line is the actual value. The horizontal ordinate is the first 60 sets of data for 400 sets of testing sets, and the vertical ordinate is the position value. The solid line and the dashed line are basically completely and the error loss approaches 0, which indicates that the predicted value of the BO-LSTM neural network is accurate and that expected effect is realized.

#### 5.2.2. Performance Analysis Of Intelligent Physics Engine.

The results after training the BO-LSTM neural network can only show that the movement demand of the flexible manipulator is satisfied by the prediction accuracy, but it cannot fully embody the advantages of BO-LSTM neural network in solving inverse kinematic. Therefore, the accuracy and speed performance of the intelligent physics engine based on BO-LSTM neural network are tested. The experimental hardware used is an 11th Gen Intell i7-11800H CPU with 32GB of RAM.

In terms of accuracy performance, the intelligent forward physical engine of the BO-LSTM neural network is trained and tested 20 times, and the MSE, MRE, MAE, R^2^ and CI width are calculated. The BO-LSTM neural network is compared with the BP neural network, RBF neural network, and the LSTM neural network, and the results are shown in Table 2. The number of hidden layers, the number of epochs, the error threshold and the learning rate of BP neural network is 10, 1000, 1e-6 and 0.1, respectively. The number of RBF units and the spread of RBF neural network is 6 and 5, respectively. The batch size, the number of neurons in the LSTM layer, the learning rate and the number of epochs is 600, 10, 0.001 and 500, respectively.

The accuracy of the BO-LSTM neural network is much higher than the BP, RBF, and LSTM neural network, as shown in Table 2. On the one hand, the BO-LSTM neural network model has unique advantages in fitting of long-term data; on the other hand, there is an over-fitting problem between the BP neural network and the RBF neural network. When the prediction limit is reached, the prediction effect decreases with increasing training time.

**Table 2:** Interval setting of BO-LSTM hyperparameters. Comparison of MSE, MRE, MAE, R^2^ and CI width between BP, RBF, LSTM and BO-LSTM neural network. Note: [BP, RBF, LSTM, BO-LSTM].

	Forward database	Inverse database
MSE	[7.407, 7.117, 5.595, 0.065]	[0.183, 0.141, 0.062, 0.056]
MRE	[0.153, 0.141, 0.102, 0.023]	[0.053, 0.042, 0.028, 0.025]
MAE	[4.246, 4.135, 1.918, 0.312]	[0.265, 0.213, 0.154, 0.137]
R^2^	[0.852, 0.874, 0.926, 0.992]	[0.921, 0.944, 0.988, 0.998]
CI width	[2.174, 1.255, 0.399, 0.239]	[0.066, 0.045, 0.021, 0.015]

**Fig 18:**
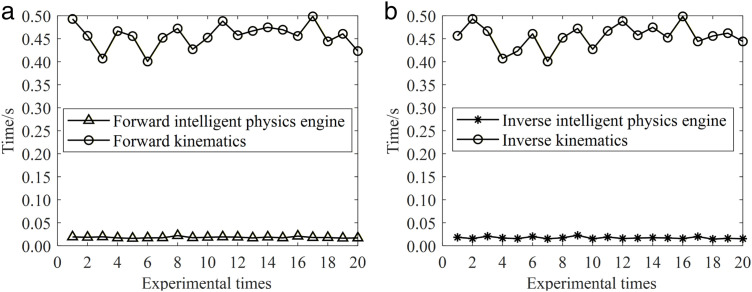
Time consumption comparison of inverse solution of continuous manipulator. (a) Forward physics engine. (b) Inverse physics engine.

In terms of efficiency performance, the end pose of the same continuous manipulator is input, the time needed to obtain 8 joint angles by the three methods is recorded, and 20 experiments are carried out. The comparison is shown in Fig 18. The roundness is the time consumption of the inverse kinematics solution, the triangle is the time consumption of the forward intelligent physics engine, and the asterisk is the time consumption of the inverse intelligent physics engine. The time consumption of the numerical method can be obtained by the weighted average of 20 experiments, which is 0.501 s. The forward intelligent physics engine takes 0.018 s and the inverse intelligent physics engine takes 0.017 s. Therefore, the intelligent physics engine improves the computational efficiency by more than 27 times.

The control period of the hyper-redundant cable-driven flexible manipulator is 0.05 s, which means that the control system needs to complete the refresh of the control signal within 0.05 s. The forward intelligent physics engine and the inverse intelligent physics engine proposed in this paper show efficiency advantages in the solution process, only 0.018 s and 0.017 s respectively. Therefore, the intelligent kinematic physics engine meets the real-time control requirements of the hyper-redundant cable-driven flexible manipulator, and provides a strong technical support for the precise operation and efficient operation of the manipulator.

### 5.3. Simulation experiment

In order to verify the effectiveness of the intelligent kinematics physics engine, the SimMechanics module in MATLAB is used to simulate the hyper-redundant cable-driven flexible manipulator. SimMechanics is a module in MATLAB for mechanism simulation, which can define different types of joints and connecting rods to construct the physical structure of the manipulator and automatically calculate the kinematics of the manipulator, and visualize the simulation results of the manipulator. Whether the prediction result of the intelligent kinematics physics engine conforms to the motion of the manipulator can be verified by simulation, so that the effectiveness of the BO-LSTM neural network is verified.

Unified Robot Description Format (URDF) is a file format used to describe the characteristics of manipulators, which can express parameter information such as kinematics and dynamics of manipulators, and quickly model manipulators. The size, appearance, material, color and other properties of the manipulator can be compiled by URDF. URDF is usually used to describe regular robots, and complex robots is difficult to describe directly with URDF. To solve this problem, the SolidWorks provides the sw_urdf_exporter plug-in, which can assist in converting models designed in SolidWorks into URDF format files. In order to ensure that the dynamic characteristics of the simulation model are highly consistent with the actual manipulator, the material and dynamic parameters of each component are set in detail when designing the hyper-redundant cable-driven flexible manipulator model in SolidWorks. Specifically, the joint and universal joint materials of the manipulator are 304 stainless steel, the mass of each joint is 1 kg, and the mass of the universal joint is 0.2 kg. In addition, in order to simulate the friction effect in the actual movement, the friction coefficient of the joint and the universal joint is set to 0.01. The value is within the range of the friction coefficient of common metal materials, which can truly reflect the friction of the manipulator during the movement.

The creating process of the URDF format file of the hyper-redundant cable-driven flexible manipulator is divided into three steps. Firstly, the rotation axis of each joint is established by the rotation position of each joint of the manipulator, and the position and direction of each joint coordinate system are set by the D-H coordinate system of the manipulator in Fig 6. Secondly, the kinematic link of the manipulator is configured, the connection relationship between joints is defined, and the correct coordinate system and rotation axis are selected. Finally, the URDF folder of the hyper-redundant cable-driven flexible manipulator is generated.

**Fig 19:**
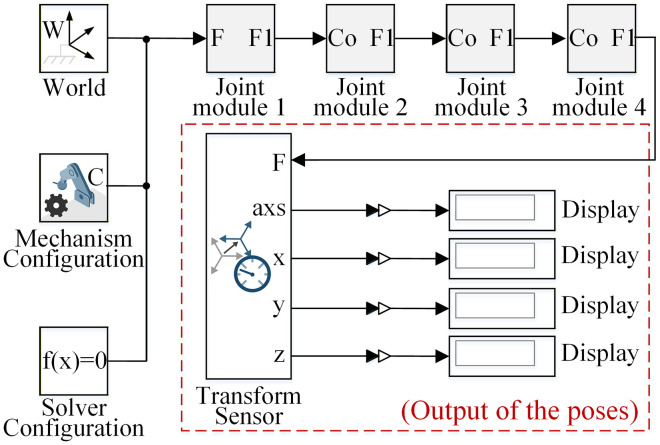
Simulink model of hyper-redundant cable-driven flexible manipulator.

**Fig 20:**
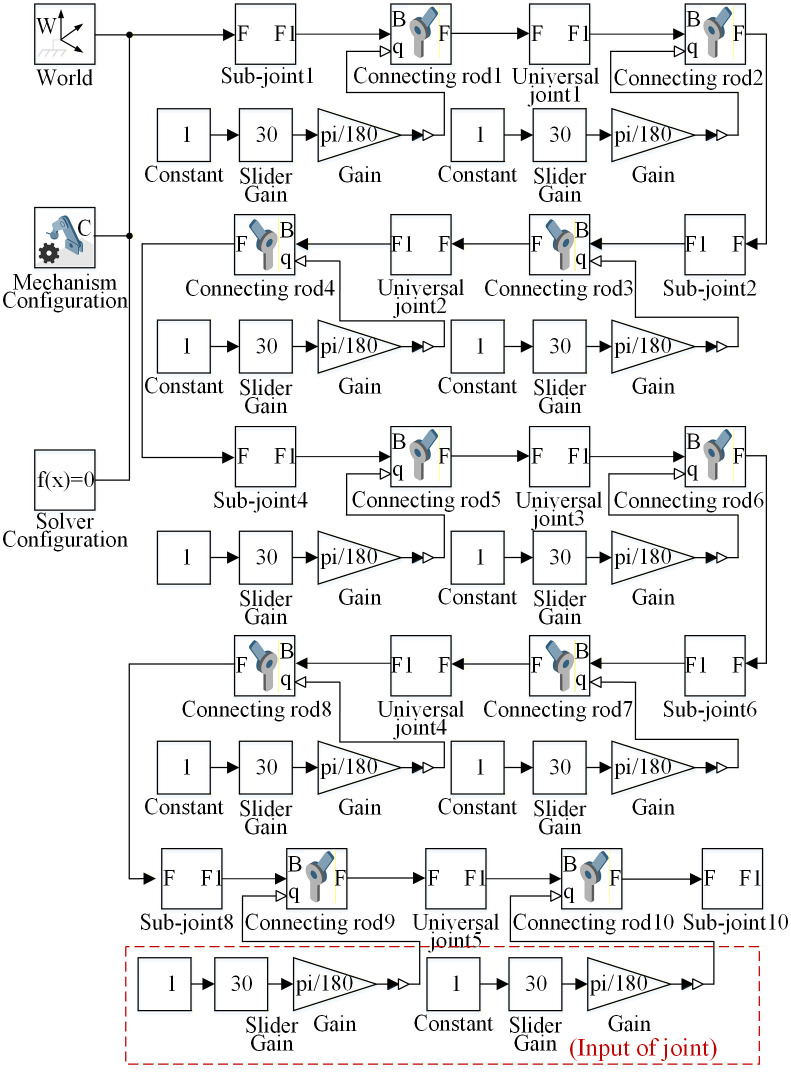
Simulink model of first joint module.

**Fig 21:**
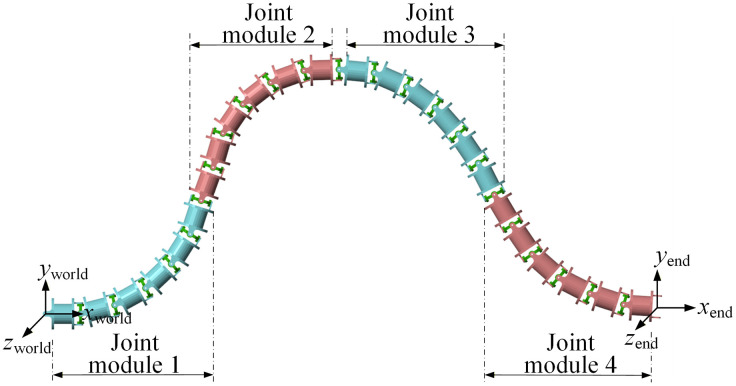
Simulation interface of hyper-redundant cable-driven flexible manipulator.

The URDF file is imported into the SimMechanics for the manipulator motion simulation after the URDF file of the manipulator is created. Firstly, the model is imported into simulink, and joint angle input modules and pose output modules are added respectively. The simulink model is shown in Fig 19. In order to further explain the connection situation of the joint module, the joint module 1 is expanded as shown in Fig 20. Secondly, the simulation speed regulation is selected, and the speed regulation is enabled to achieve the purpose of real-time control of the joint angle. Thirdly, a real-time script is created in matlab to start SimMechanics. Finally, the model is run and the correct angle information is input to obtain the end pose of the manipulator. The simulation interface is shown in Fig 21. In the simulink model of the manipulator, the world module is the world coordinate system. The mechanism configuration module specifies the overall gravity and simulation parameters of the manipulator system. The solver configuration module configures and controls the parameters of the simulation solver to ensure the accuracy and efficiency of the simulation. The sub-joint module simulates the sub-joints in the actual manipulator. The universal joint module simulates the universal joint in the actual manipulator. The connecting rod module represents the connection and force transmission between the sub-joint and the universal joint, which is used to simulate the dynamic behavior of the manipulator. The constant module provides an initial value, which is the starting point of the slider gain module. The slider gain module can dynamically change the angle output, which is used for dynamic simulation of the manipulator. The Gain module converts angle signal into radian signal. The transform sensor module measures the coordinate transformation information of the manipulator. The display module is used for real-time signal display.

**Fig 22:**
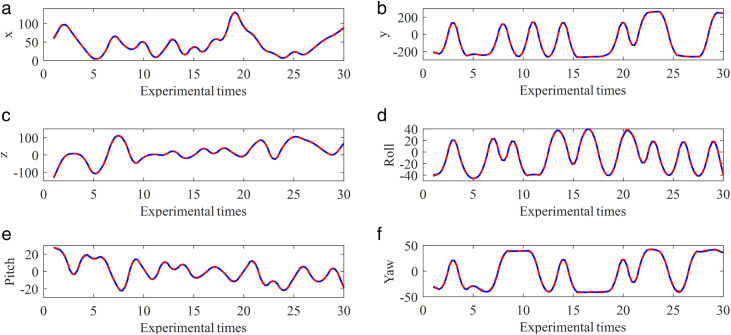
Comparison of simulation values and predicted values for position and orientation of manipulator. (a) Position *x*. (b) Position *y*. (c) Position *z*. (d) Orientation roll. (e) Orientation pitch. (f) Orientation yaw.

The error between the predicted results of the intelligent kinematics physics engine and the simulation results of the SimMechanics manipulator is compared by 30 sets of joint angles to verify the effectiveness of the intelligent kinematics physics engine. The position and orientation of the hyper-redundant cable-driven manipulator are shown in Fig 22. The blue solid line and the red dash line shown in Fig 22 represent the simulation results of SimMechanics and the prediction results of the intelligent physical engine, respectively. The prediction accuracy of the intelligent kinematics physics engine is 98.6%, which meets the requirements of the kinematics solution of the hyper-redundant cable-driven flexible manipulator.

## 6. Conclusion

In this paper, the problem that the analytical model of a hyper-redundant cable-driven flexible manipulator is difficult to establish are solved. Firstly, the forward and inverse solution model of the hyper-redundant cable-driven flexible manipulator are established by using the hierarchical method. The highly nonlinear inverse solution model is normative analyzed by using the Newton-Euler method, and a high-precision numerical inverse solution model is established. Secondly, considering the real-time problem of the numerical inverse solution model of the hyper-redundant cable-driven flexible manipulator, based on the analytical forward solution model and the high-precision numerical inverse solution model, the forward database and the inverse database based on the Monte Carlo method are established. Finally, in the BO-LSTM neural network, an inverse solution physics engine is established based on the forward database and the inverse database respectively, which significantly improves the computational efficiency of the hyper-redundant cable-driven flexible manipulator, and has higher computational accuracy than other neural network models. The forward and inverse solution model, high-precision numerical inverse solution model and intelligent physical engine of the hyper-redundant cable-driven flexible manipulator established by the hierarchical method proposed in this paper have modular characteristics. The manipulator can be easily assembled and expanded when the degree of freedom of the hyper-redundant cable-driven flexible manipulator is increased, which provides the necessary theoretical basis for the development of the hyper-redundant cable-driven flexible manipulator and provides important technical support for real-time path planning and arm-shaped planning.

In the future research, the research on model generalization ability, dynamic modeling and control strategy will be further expanded on the basis of the kinematic model of the hyper-redundant cable-driven flexible manipulator. More optimization strategies will be explored to improve the scalability of the BO-LSTM model to adapt to the complex dynamic characteristics of the hyper-redundant cable-driven flexible manipulator. The dynamic characteristics of the manipulator will be deeply analyzed, and an accurate dynamic model will be constructed to accurately capture the dynamic response characteristics under different motion states, which will provide rigorous theoretical support for the design of subsequent control algorithms. At the same time, the advanced control methods such as model-based adaptive control, robust control and data-driven intelligent control will be explored and developed to significantly improve the motion accuracy and stability of the manipulator and ensure the reliability and adaptability in complex task environments. Finally, the real-world experiments will be carried out to verify the effectiveness and reliability of the developed model and control strategy, and to ensure the superior performance of the hyper-redundant cable-driven flexible manipulator in practical engineering applications.
